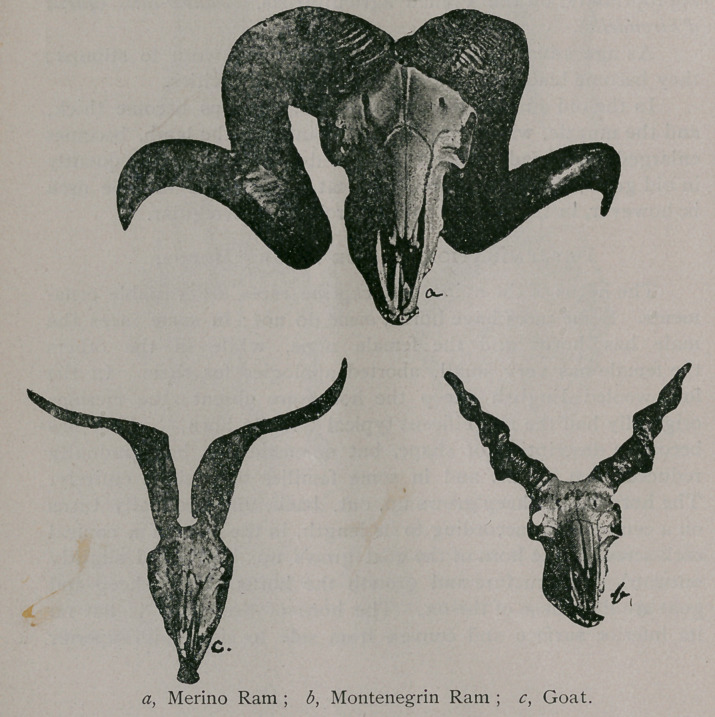# Age of Sheep and Goat

**Published:** 1891-11

**Authors:** R. S. Huidekoper

**Affiliations:** Vet.


### AGE OF THE SHEEP AND GOAT.*

The sheep is of value for any purpose for a still more limited period than the ox, and its exact age is of less importance, except in the case of valuable breeding animals, which are always possessed of a registered pedigree which guarantees the day of their birth. An approximation within a month or two is sufficient for practical purposes in a spring lamb, and an error of six months will not alter the taste of a four-year-old South-down wether for a roast saddle. After this time it is a poor economist, except in districts where wool is the only product to be derived from the sheep, who will not turn it over to the butcher,— again excepting the pedigreed breeding animal. Civilization, better agriculture, and the care of man have altered the physiological characters of the ovine races even more than they have those of the bovine, and a larger percentage of the former have been rendered precocious than of any other species of animal. As a result of the improved agricultural needs, the sheep has altered considerably in form; the eruption of the teeth is more hasty, the

* From advance sheets of AGE of the DOMESTIC ANIMALS, by R. S. Huidekoper, M. D. Vet.

horns have diminished in size or are absent, and the general shape of the animal has been modified.

The age of the sheep is determined by the character of the teeth, and of the horns, when the latter are present:

DENTITION.

$$\text{Formula}\begin{cases}\text{Temporary, . . . .} & \dfrac{0\,.\,0\,.\,3}{4\,.\,0\,.\,3} = 20\\[2ex] \text{Permanent, . . . . .} & \dfrac{0\,.\,0\,.\,6}{4\,.\,0\,.\,6} = 32\end{cases}$$

The sheep has thirty-two teeth like the ox, eight incisors in the lower jaw, none in the upper, six molars in each arch of either jaw, making twenty-four molars in all, and no tushes.

There sometimes exist small supplementary premolars.

INCISORS.—The incisors are eight in number. They are set firmly in their alveolar cavities in the maxilla, and form an arch more convex than either the incisive arch of the horse or the ox. As in the ox, they are termed the *pincher*, *first intermediate*, *second intermediate*, and *corner* teeth. As in the other animals, there are two sets, the *temporary* incisors and the *permanent* incisors. The temporary incisors are much smaller and proportionately much narrower than the permanent ones; so that the jaw of the lamb has an elongated, narrow appearance, which alters greatly in form, becoming wider and more flat, in the older animals. The incisors of both dentitions are wedge-shaped like the permanent incisors of the horse. They have no neck separating the crown from the root, like the incisors of the ox and the temporary incisors of the horse. They are firmly imbedded in their alveolar cavities, which allows them to nip the short grass close to the roots, and obtain a living, where the ox, with its loose incisors, can no longer obtain a hold to tear up the blades. In the virgin tooth the external face is white and polished except near the root, where it is surrounded by a black cement. The internal face has two longitudinal gutters divided by a little crest. The former are filled with black cement. They represent by their convex anterior face, in profile, the quarter of a circle. From this position they meet the cushion of the upper jaw by their free extremity, like the incisors of a young horse, and not by the posterior surface, as in the ox; so that by use they rapidly wear the anterior border and form a table to the teeth like the soliped, and not like that of their closer relation, the large ruminant. The incisors of the sheep are formed of dentine, surrounding a pulp-cavity which becomes filled with a darker-colored deposit at an early period, and are covered with a layer of enamel, which disappears toward the roots, and which is covered on the sides, in the longitudinal gutters, and near the gums by a black cement.

MOLARS.—The molars, except for their smaller size, resemble those of the ox. They gradually increase in size from the first to the sixth molar, the first three occupying but a third of the arch. The relative density of the dentine and the convoluted enamel differs greatly, so that the dentine is more rapidly worn and the enamel stands in sharp ridges from the table of the teeth.

### ERUPTION OF THE TEMPORARY TEETH.

INCISORS.—Considerable diversity of description is found among the older writers as to the time of eruption of the teeth in the sheep from the fact that some studied them in the common races of Central Europe, while others examined them only in the perfected races of England At the present day the studies of the latter will be found more acurate, as few flocks can be found which are not well bred or mixed more or less with the finer races.

At birth, the lamb may have the pinchers and first intermediate teeth through the gums, or the anterior borders of these teeth may show a whitish line where they press against the gum, which they pierce in from three to five days. The second intermediate emerges from the gum about the tenth day, and is followed tardily by the corner teeth, which do not appear until the twenty-eighth or thirtieth day.

MOLARS.—The three temporary molars in each arch appear about the third week, when we find the first six incisors sufficiently advanced from the guns to allow the young animal to nip herbage, which is to be ground by larger teeth.

### ALTERATIONS DURING THE FIRST YEAR.

*One to Three Months.*—During this time there is little change in the teeth. They are slightly more prominent, but retain their virgin appearance.

*About Three Months.*—Sometimes just before and sometimes just after three months from the birth of the animal the fourth (first permanent) molar appears, that of the lower jaw preceding that of the upper by a week or more.

*Three to Nine Months.*—During this half year no characters of substantial value can be found. The incisors during this period attain their full size, and, according to the herbage or other food on which the animal has been fed, more or less wearing of the anterior borders of the teeth has taken place. In addition, however, to the general aspect of the animal, it is easy to recognize the difference between the fresher teeth, which have scarcely been worn, of a lamb four to five months of age, and the worn, broken, and loosening ones at eight, nine, or ten months.

*Nine Months.*—At nine months the fifth (second permanent) molar makes its eruption. This fixes an important land-mark in the determination of the age of the young animal.

The fourth and fifth molars are formed of two principal lobes, each with two points of enamel, so that there are four points of enamel; the internal points are higher on the lower teeth, while the external ones are longer on the upper teeth. These points become used, and as they are worn away the band of central enamel is separated from the outer border of the teeth by a larger area of dentine.

The character of the points of enamel on these teeth, or the distance of the bands of enamel from the outer border of the teeth, thus indicates whether the animal has just passed nine months or is a year or more old.

*Ten Months.*—At about ten months the body of the maxilla is seen to be wider and broader, due to the development of the permanent pinchers, which press upon the roots of the temporary pinchers, producing absorption, and these latter are found less solidly fixed in their alveolæ.

### Eruption of Permanent Teeth.

*One Year to Fifteen Months* (Fig. I).—At this period the permanent pinchers make their appearance. In the more precocious races the eruption of the pinchers takes place at one year, while in the common races it may not until fifteen months. Again, a question of individual development may hasten or retard the eruption by a month or two; so that the better-bred animal may be thirteen or fourteen months before the first two permanent teeth have appeared, while the more hasty, common animal may have its permanent teeth by the same time. In the male the eruption takes place very slightly earlier than in the female.

The conditions of the points on the fourth and fifth molars alluded to above will aid in deciding if the eruption has been hasty or tardy.

*Eighteen Months* (Fig. II).—At a year and a half the first intermediate permanent incisors and the six permanent molars appear. The eruption of these teeth is followed very shortly by the falling out of all the temporary molars, which are replaced almost simultaneously by the permanent molars. After two years the molars furnish but little indication of the age. From this time, according to its sex, the animal takes the name of *ram*, or *ewe*.

*a.* External Face. *b.* Internal Face.

*Two Years and Three Months* (Fig. III). About nine months after the eruption of the first intermediate teeth the second *intermediate* appears ; whether in prococious or tardy races, the interval between the eruption of the first and second iutermediate teeth is some three months longer than that between the former and the pincher teeth. In tardy animals, in which the first intermediate have not appeared until they are nearly two years of age, the second intermediate may be delayed until two years and nine months.

*a.* External Face. *b.* Internal Face.

*Three Years* (Fig. IV).—At three years the eruption of the corner teeth takes place, although under the conditions given above certain animals may be several months late in completing their dentition.

A greater irregularity is found in regard to the appearance of the corner teeth than in the sequence of eruption of the others. By the time of the appearance of the corner teeth the pinchers and first intermediate may become worn and even leveled. Girard first noted that some sheep have but six incisors, the corner teeth remaining aborted under the gum.

*After four years* (Fig. V) there is a continuous but very irregular wearing of the teeth, which is governed by the nature of the food. Those animals which are fed on fine herbage and those which are stall-fed level off the tables evenly until but stumps remain; but in all sheep there is always more rapid wearing of the pinchers and first intermediate teeth, which gives the plane of the tables of the arch a concave form.

Those animals fed on the hill-sides and where shrubs and undergrowth are abundant use their teeth much more rapidly, and wear notches between them like the markings on a horse, which cribs on a vertical object; these notches are called very appropriately, by the French agriculturists, *swallow-tails* (*queue d'hirondelle*).

As age advances the teeth of the sheep are worn to stumps; they become black and loose in their alveolar cavities.

In the old animals the face wrinkles, the lips become thick, and the muzzle, which was fine and pointed in the lamb, becomes enlarged and broad. Sometimes in old sheep, but more frequently in old goats, the incisors attain a great length; the incisive arch is, however, in these cases usually broken and irregular.

### Determination of Age by the Horns.

The horns of the ovine and caprine races are variable ornaments. Some races have horns, some do not; in some races the male has horns and the female none, while in the others the female has very small, aborted apologies for them. In the long-wooled English sheep the horns are absent; the merinos originally had the magnificent typical "ram's horn," which has become a descriptive of shape, but domestication has gradually reduced them in size, and in some families they abort entirely. The horn of the sheep grows up, out, back, and gradually turns on a central axis, according to its length, in the form of a conical cork-screw. The horn of the goat grows up, back, and slightly outward. In structure and growth the horns of the sheep and goat are like those of the ox. The horn of the sheep is flat on its inferior surface and convex from side to side on its superior surface ; it is divided transversely by ridges ; the interspaces have longitudinal scaly ridges and gutters.

The horns appear about fifteen days after birth, attain their greater growth during the first year, and cease growing after four years. If the animal is castrated the growth of the horn lessens, whereas in the ox castration stimulates the growth.

The epidermic covering, which extends from the skin on the appearance of the horn, dries and scales off in six weeks to two months, leaving a roughened, scaly surface. The size and roughness of these series of scaly sections vary from year to year, and indicate the years until the horns cease to grow,—at four. Observations by Girard on the growth of the horns of merino rams (the horns of the merinos are the most typical) showed the growth to be :—

The first year, 19 to 20 inches ; the second year, 5 to 6 inches; the third year, 3 to 4 inches ; the fourth year, 2 to 3 inches ; —in all, 29 to 33 inches.

## Figures and Tables

**Figure f1:**
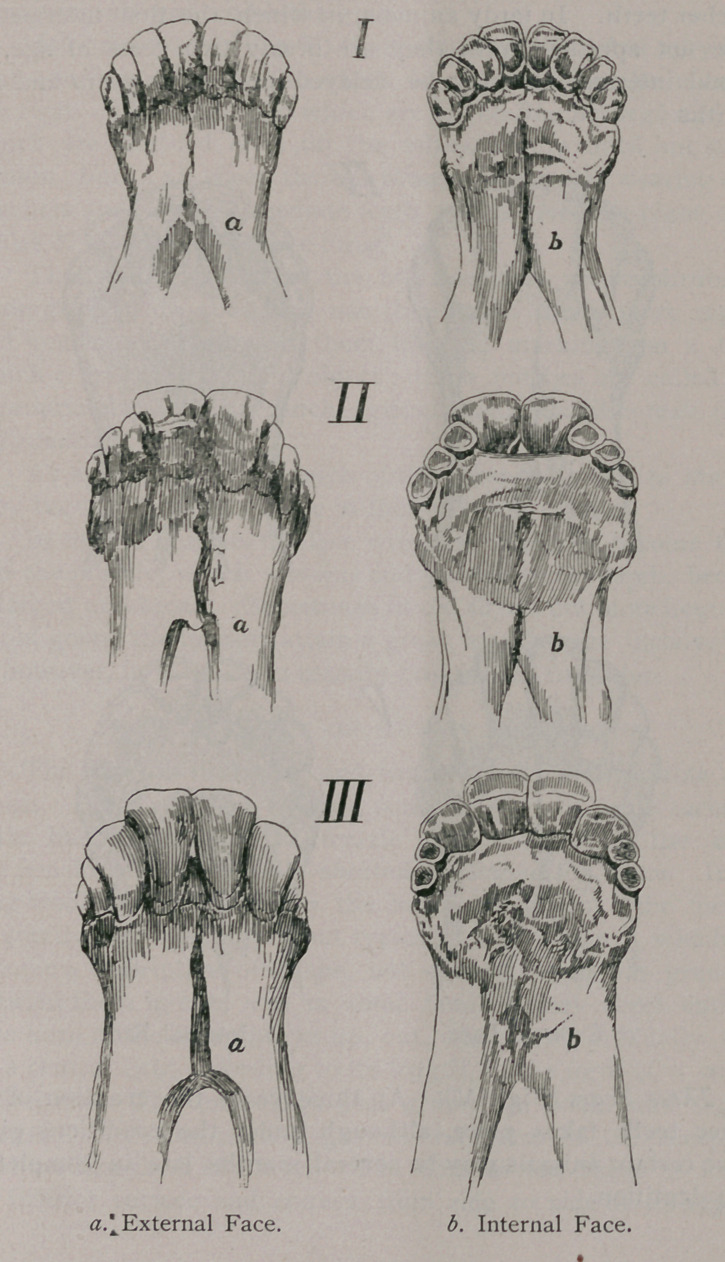


**Figure f2:**
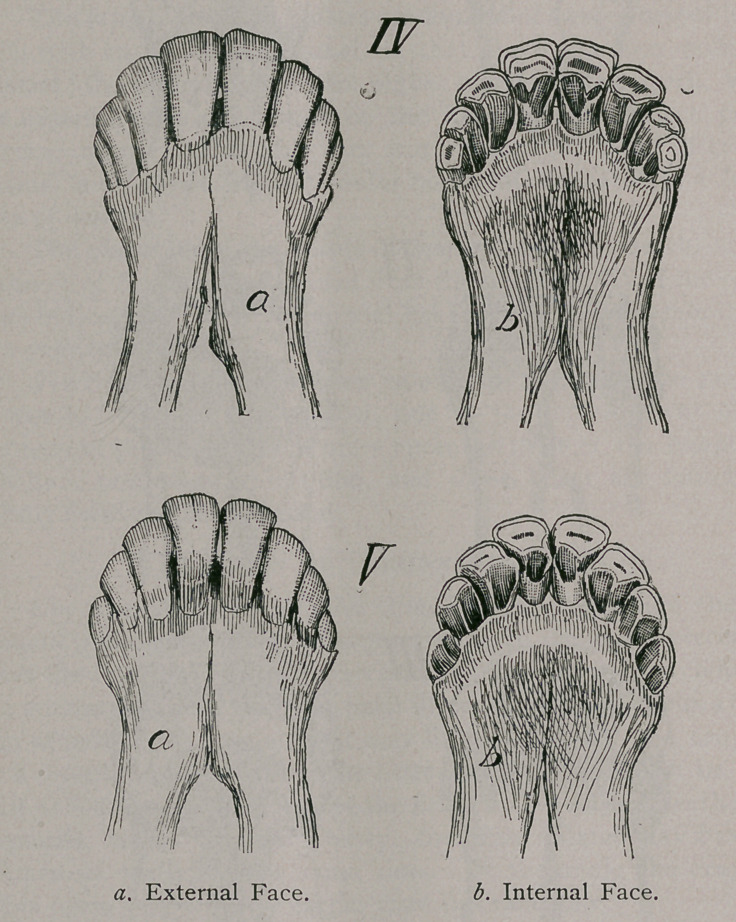


**Figure f3:**